# Response of PIP aquaporins to long-term cold stress in two citrus rootstocks

**DOI:** 10.1371/journal.pone.0355050

**Published:** 2026-07-31

**Authors:** Amparo Primo-Capella, Mary-Rus Martínez-Cuenca, Emma García-Hiltner, María Ángeles Forner-Giner

**Affiliations:** Department of Citriculture and Vegetal Production, Valencian Institute for Agricultural Research–IVIA, Moncada, Valencia, Spain; Birla Institute of Technology & Science Pilani, K. K. Birla Goa Campus, INDIA

## Abstract

Cold is one of the most impactful abiotic stresses causing significant crop losses. Additionally, it is a major limiting factor, reducing cultivation areas, especially for tropical and subtropical species such as citrus. In this study, we conducted a physiological assessment of water balance, photosynthesis, fluorescence, and transcriptomic data, to further understand the response of aquaporins in the Valencia Delta Seedless variety grafted onto two rootstocks: *Citrus macrophylla* and Carrizo citrange, considered sensitive and tolerant rootstocks, respectively. After 6 weeks of exposure to 1°C with a photoperiod of 16 hours of light and 8 hours of darkness, measurements were taken of CO_2_ assimilation rate, stomatal conductance, substomatal CO_2_ concentration, transpiration rate, and photosynthetic apparatus damage (Fv/Fm). In addition, plant water balance was quantified by measuring water potential (Ψw), osmotic potential (Ψπ), and relative water content (RWC%). Finally, gene quantification was performed for three previously selected aquaporins (*PIP1–2, PIP2–2, and PIP2–5*), which our laboratory has formerly associated with cold acclimation and, consequently, with tolerance. Cold temperatures lead to stomatal closure, reduced transpiration, and a significantly decreased osmotic potential (Ψπ), like what occurs under conditions of extreme drought, as the plant seeks to prevent water loss through transpiration. However, the stem water potential (Ψw) and relative water content (RWC) often remain at levels typical of actively growing plants under adequate irrigation conditions. Gene expression and physiological results suggests that plants grafted onto Carrizo citrange rootstock exhibit a better cold response and increased acclimation, leading to the overexpression of aquaporins *PIP1–2*, *PIP2–2*, and *PIP2–5*.

## Introduction

Cold stress is one of the primary abiotic factors causing significant crop losses and limiting cultivation areas. Citrus crops, originating from tropical and subtropical climates, are particularly sensitive to cold. Currently, climate change is altering weather patterns, which implies that cultivation zones for certain species may shift and change in the foreseeable future. Therefore, understanding the thresholds of popular species is of great importance.

In the Mediterranean region, the rootstock *Citrus macrophylla* Wester is widely used for grafting with lemon trees to confer tolerance to tristeza virus (CTV). However, this vigorous rootstock is highly sensitive to low temperatures [1–4] and exhibits some sensitivity to when grafted with other varieties. Thus, for the purposes of this study, *C. macrophylla* is an ideal material to consider as a cold-sensitive rootstock. On the other hand, *Poncirus trifoliata* (L.) Raf. is the most cold-tolerant known citrus rootstock [3,5,6]. However, its high sensitivity to iron chlorosis has led to its disuse in the Mediterranean region, where soils are extremely calcareous [7–10]. In its place, the Carrizo citrange [*Citrus sinensis* (L.) Osbeck × *Poncirus trifoliata* (L.) Raf.] rootstock is widely used, along with other Forner-Alcaide hybrids [8,9,11–14]. Following extensive low-temperature studies on these citrus genotypes within our research group, we have demonstrated that the rootstock influences variety tolerance, establishing it as a heritable trait, consistent with findings for other abiotic stresses [10,12,15–17]. In a previous investigation employing the same genotypes under long-term cold stress, it was observed that aquaporins may play a significant role in the low-temperature tolerance of citrus [16], all PIP-type aquaporins were suppressed in both rootstocks, with the exception of three genes that exhibited a distinct expression pattern characterized by significant overexpression (Data not published). The specific PIPs demonstrating this behavior were PIP1−2 (LOC18048648), PIP2−2 (LOC18039157), and PIP2−5 (LOC18043954), as they are implicated in the regulation of transcellular water movement. Water transport within a plant occurs through three main pathways: (1) apoplastic transport, which occurs along cell walls without crossing cell barriers, allowing for long-distance water movement along the water potential gradient; (2) symplastic transport, which takes place between cells through plasmodesmata; and (3) transcellular transport, across cell membranes [18]. Under abiotic stress conditions such as drought, salinity, or temperature extremes, plants face a trade-off between water consumption for essential physiological functions, such as CO_2_ fixation, water photolysis, and transpiration, or conserving resources by halting growth. Long-distance apoplastic transport is the primary pathway through which plants distribute water across different tissues. The plant’s water status is typically characterized by water potential (Ψw) and relative water content, RWC% (Relative Water Content, as a percentage of maximum hydration) [19]. Flexas and Medrano (2002) define three levels of water stress and these values vary by species [20].1. Mild water stress: reduction of Ψw by several bars or reduction of RWC% to 100−85%, 2. moderate water stress: reduction of Ψw to 12−15 bars or reduction of RWC% to 85−70%, 3. severe water stress: reduction of Ψw below 15 bars or reduction of RWC% below 70%.

Aquaporins belong to a highly conserved group of membrane-bound proteins known as Major Intrinsic Proteins (MIPs). As sessile organisms, plants lack a circulatory system; their cells contain numerous organelles that require precise regulation of water to adapt to environmental fluctuations. Aquaporin gene families vary significantly across species, ranging from 31 in maize to 71 in cotton, with sweet orange containing 34 members [21–31]. MIPs are classified into different families based on their expression patterns, amino acid sequence modifications, regulatory mechanisms, and intracellular localization [21–23].

The maintenance of plant turgor pressure and hydraulic homeostasis is critically dependent on non-vascular, transcellular water transport. Turgor pressure is vital at multiple organizational levels: it supports cellular processes like metabolism and growth, regulates stomatal aperture at the tissue level, and influences organ movement and overall plant water status [23]. PIPs facilitate the transport of water, glycerol, hydrogen peroxide, carbon dioxide, and urea. PIPs are also known to participate in responses to abiotic stresses such as salt, boron, drought, and low temperatures [21,24].

In this study, we conducted a physiological and gene quantification analysis on the role of previous selected aquaporins in two citrus species relevant to citriculture, *C. macrophylla* and Carrizo citrange, representing cold-sensitive and cold-tolerant genotypes, respectively grafted with Valencia delta seedless. Our objective was to elucidate the role of these aquaporins and water relations in the cold tolerance of citrus plants and the rootstock influence. The physiological and gene expression results presented here, together with other findings from our research group, lead us to propose that varieties grafted onto low-temperature-tolerant rootstocks achieve acclimation faster than those grafted onto sensitive rootstocks. This accelerated acclimation may be the underlying mechanism for their enhanced cold tolerance.

## Materials and methods

### Plant material and growth conditions

Eighteen-month-old plants of the Valencia delta seedless variety grafted onto Carrizo citrange [*C. sinensis* (L.) Osbeck. x *Poncirus trifoliata* (L.) Raf.] and *C. macrophylla* were obtained from a nursery (Viveros Sevilla SA, Sevilla/Tocina road km 147, 41310, Brenes, Spain) and no permission was necessary to collect the plants. Plants were grown individually in 4-liter opaque plastic pots filled with substrate composed of peat, coconut fiber, sand and perlite (40:25:25:10). Plants were irrigated twice weekly with the following basal nutrient solution (pH 6.0) at half strength: 5 mM Ca(NO_3_)_2_, 1.4 mM KNO_3_, 2 mM MgSO_4_, 0.6 mM H_3_PO_4_, 20 µM Fe-EDDHA, 7.6 µM ZnSO_4_·7H_2_O, 0.50 µM CuSO_4_·5H_2_O, 50 µM H_3_BO_3_, 0.50 µM MoO_3_ and 54 µM MnSO_4_·H_2_O. Plants were acclimated for 2 weeks before the experiments began under greenhouse conditions (26–28/16–18°C, 70–80% and a 16-hour photoperiod).

Plants were selected according to their size uniformity. The control group comprised twelve plants, six for the Carrizo citrange genotype and six for *C. macrophylla*. It was left under controlled greenhouse conditions, which were the same conditions before experiments began: 26–28/16–18°C temperature, 70–80% humidity and a 16-hour photoperiod (16 h light/8 h dark). The cold-treated plants were formed by thirty-six plants: eighteen plants for Carrizo citrange that were sampled at 2, 4 and 6 weeks (six plants per each time point) and eighteen for *C. macrophylla* that were sampled at 2, 4 and 6 weeks (six plants per each time point). The cold plants were cultured in a Versatile Environmental Test Chamber (MLR-350, Sanyo) with a temperature range from 1ºC to 2ºC, both day and night, and a 16-hour photoperiod and 8 h of darkness (500 μmol m^−2^ s^−1^, 400–700 nm). Relative humidity was maintained at approximately 80%. Irrigation frequency for cold-treated plants was reduced to once per week, since, owing to the long-term nature of the trial and the concomitant decrease in transpiration, water requirements were lower, thereby reducing the risk of waterlogging-induced stress.

### Photosynthetic activity

The net CO_2_ assimilation rate (ACO_2_, μmol CO_2_ m^-2^ s^-1^), transpiration rate (E, mmol H_2_O m^-2^ s^-1^), substomatal CO_2_ concentration (Ci, μmol CO_2_ mol^-1^) and stomatal conductance (gs, mmol H_2_O m^-2^ s^-1^) of single attached leaves were measured between 10 am and 11.30 am, which allowed measurements to be taken under relatively stable conditions. Photosynthetically active radiation (PAR) on the leaf surface was adjusted to a photon flux density of 1,000 μmol photons m^-2^ s^-1^. Closed gas exchange (CIRAS-2, PP-systems, Hitchin, UK) was used for measurements. Leaf laminae were fully enclosed within a PLC 6 (U) universal leaf autocuvette in a closed circuit model and were kept at 25 ± 0.5 °C with a leaf-to-air vapour deficit of about 1.7 Pa. The air flow rate through the cuvette was 200 mL min^-1^. Measurements were taken weekly on the two youngest fully expanded leaves on all six trees for 6 weeks. The average value of the two leaves was considered representative of each individual plant.

### Leaf water relations

Leaf water potential (was measured with a Scholander-type pressure chamber (Soilmoisture Equipment Corp., Santa Barbara, CA, USA). After measurements, two expanded leaves were tightly wrapped in aluminium foil for 1 hour, measured (Ψω), frozen by immersing in liquid nitrogen and stored in a freezer at −80 °C. After thawing, the leaf osmotic potential (Ψπ) was measured in the extruded cell sap collected at 25 ± 1 °C and placed inside an osmometer (Digital Osmometer, Wescor, Logan, UT, USA).

Similar sized leaves from each plant were weighed to determine the relative water content (RWC%) of leaves was calculated as RWC = (FW – DW) – (TW – DW) −1 x 100. Where FW was the fresh weight, DW was the drought weight and TW was the turgent weight (after hydrating the leaf for 24 hours).

In both experiments, evaluations were made using two uniform fully expanded mature leaves from the mid-stem zone for all six replicates per treatment during 6 weeks. The average value of the measurements taken on the two leaves was taken as being representative of each individual plant.

### Fluorescence measurements

CFI (Chlorophyll Fluorescence Image) (Fv/Fm) was measured at 2 weeks of the experiment in a portable fluorometer (PAM-2100 Walz, Effeltrich, Germany). Two leaves per plant were darkened for 30 min prior to taking measurements. Minimum (dark) fluorescence Fo was obtained upon excitation of leaves with a weak beam from a light-emitting diode. Maximum fluorescence (Fm) was determined following a 600 ms pulse of saturating white light. The variable fluorescence (Fv) yield was calculated as Fm − Fo. Further information on CFI (Chlorophyll Fluorescence Imagine) measurements can be obtained from [25].

### RNA isolation and gene quantification by qRT-PCR

Leaf samples were collected, placed in liquid N2 and stored at −80 °C. RNA was extracted from 100 mg of tissue using the RNase Plant Mini Kit (Qiagen, CA, USA) with the RLT-β-mercaptoethanol buffer (Merck, Burlington, MA, USA). Genomic DNA contamination was removed by the RNase-Free DNase Set Kit (Qiagen, CA, USA) from column digestion according to the manufacturer’s instructions. Purified RNA (1 µg) was used to perform the reverse transcription reaction with the enzyme (SuperScript III Life Technologies, Carlsbad, CA, USA) in a total volume of 20 µl. cDNA was diluted 50 times and 2 µl were used as a template for the RT-PCR reaction in a final volume of 20 µl. The quantitative RT-PCR reaction was performed in a StepOnePlus Real-Time PCR System thermal cycler (Life Technologies, Carlsbad, CA, USA) using Ex Taq polymerase (TliRNase H plus) (Takara Europe, SAS, Saint Germain en Laye, FR) with TB Greenpremix. The PCR program consisted of 10 min at 95 °C, followed by 40 cycles of 15 sec at 95 °C and 1 min at 60 °C. Reaction specificity was taken as the presence of a single peak on the dissociation curve and with amplified product size estimates, which was verified from agarose electrophoresis. The transcripts of genes *Citrus clementina* Actin and *Citrus clementina* UBC4, amplified with specific oligonucleotides, were used as the reference genes (Table 1) [26,27]. The calculations of the simple ANOVA and linear regression of the CT test values were taken from Brunner, Yakovlev and Strauss, 2004 to examine the variation of our reference genes. The normalization factor of the reference genes was calculated by the value of the geometric mean of both genes [28]. Relative expression was measured by the standard curve procedure with five dilution points [29]. The results were obtained with the average of the three independent biological samples with three technical replicates of each one of them.

**Table 1:** The oligonucleotides obtained for the quantification of the gene expression of the following putative genes. The annotation is shown in the format taken from NCBI, GeneBank.

GeneBank	Name	Primer sequence 5’-3’
LOC18044530	PIP1–2	FOR 5’ CCACTGATGCCAAACGGAGC 3’
		REV 5’ GACTCCTCGCTGGGTTGATG 3’
LOC18039157	PIP2−2	FOR 5’ TCACTGGAACTGGCATCAACC 3’
		REV 5’ GCTCTGAGGATGAATTGGTGGTA 3’
LOC18043954	PIP2–5	FOR 5’ GATCATCGGCACCTTTGTGTTGG 3’
		REV 5’ GAACACAGCAAATCCAATGGGGAG 3’
Reference genes		
LOC18055321	Ubiquitin	FOR 5’ TGGACGCTTCAGTCTGTTTG 3’
		REV 5’ TCGTCAATCACCCCTTCTTT 3’
LOC18037526	β-ACTIN	FOR 5’ CAGTGTTTGGATTGGAGGATCA 3’
		REV 5’ TCGCCCTTTGAGATCCACAT 3’

### Statistical analyses

For the statistical analyses, all the resulting values were the mean of six independent plants per treatment and sampled time at Control, 1 to 6 weeks. Data were submitted to an analysis of variance (multifactor ANOVA) using Statgraphics Centurion, version 16.1 (Statistical Graphics, Englewood Cliffs, NJ, USA) prior to testing for normality and homogeneity. When the ANOVA showed a statistical effect, means were separated by least significant differences (LSD) at P < 0.05.

## Results

### Long-term quantification of photosynthesis at cold temperature

**Fig 1:**
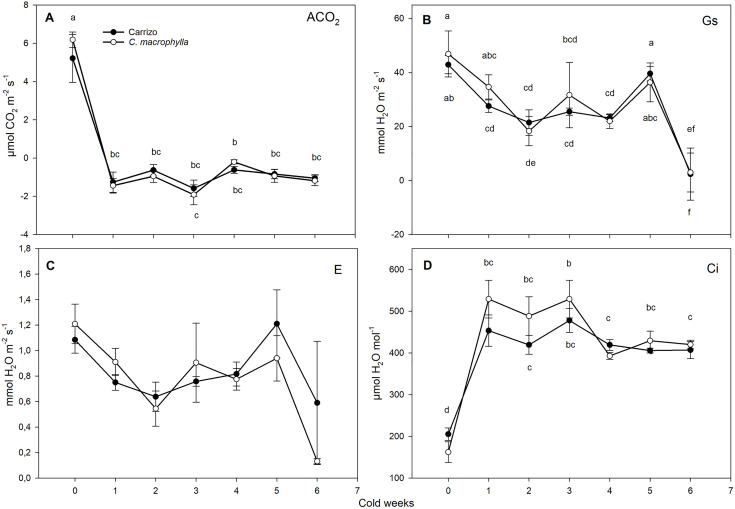
Measurement of photosynthetic activity. A) Net CO_2_ assimilation rate (ACO_2_, μmol CO_2_ m^−22^ s^−11^), B) stomatal conductance (gs, mmol H_2_O m^−22^ s^−11^), C) transpiration rate (E, mmol H_2_O m^−22^ s^−11^), and D) sub-stomatal CO_2_ concentration (Ci, μmol CO_2_ mol^−11^). Measurements correspond to Carrizo citrange and *C. macrophylla* rootstocks grafted with Valencia delta seedless and grown at 1 °C for 0, 1, 2, 3, 4, 5, and 6 weeks. The values shown represent the mean ± SD among six biological replicates (n = 6) and two technical replicates. The effect of the treatment at different times was tested with an LSD test from multiple ANOVA. The use of letters indicates significant differences, and the absence of a letter indicates ‘no significant difference’.

The physiological state of the plant, here photosynthesis, suggested that the stress being applied was very severe. Figs 1A-D showed the different indicators used to monitor photosynthesis. Fig 1A illustrated the significant decrease in CO_2_ fixation. After just one week at low temperature until the end of the experiment, the net photosynthetic rate decreased to values close to 0 μmol CO_2_ m^−2^ s^−1^ in both rootstocks. Fig 1B showed stomatal conductance (gs), which progressively fell until reaching values close to 0 mmol H_2_O m^−2^ s^−1^ after 6 weeks of treatment. Due to stomatal closure, evapotranspiration (Fig 1C) also progressively decreased until it reached values of 0.4 mmol H_2_O m^−2^ s^−1^. In the case of sub-stomatal CO_2_ concentration Ci (Fig 1D), CO_2_ concentration increased to values of 400 μmol CO_2_ mol^−1^. Overall, as previously indicated, there was a very strong repression of the photosynthesis process respect the initial/control week without showing significant differences between rootstocks at different stress times.

### Water relations, osmotic potential, water potential, and RWC% at cold temperature over the long term

**Fig 2:**
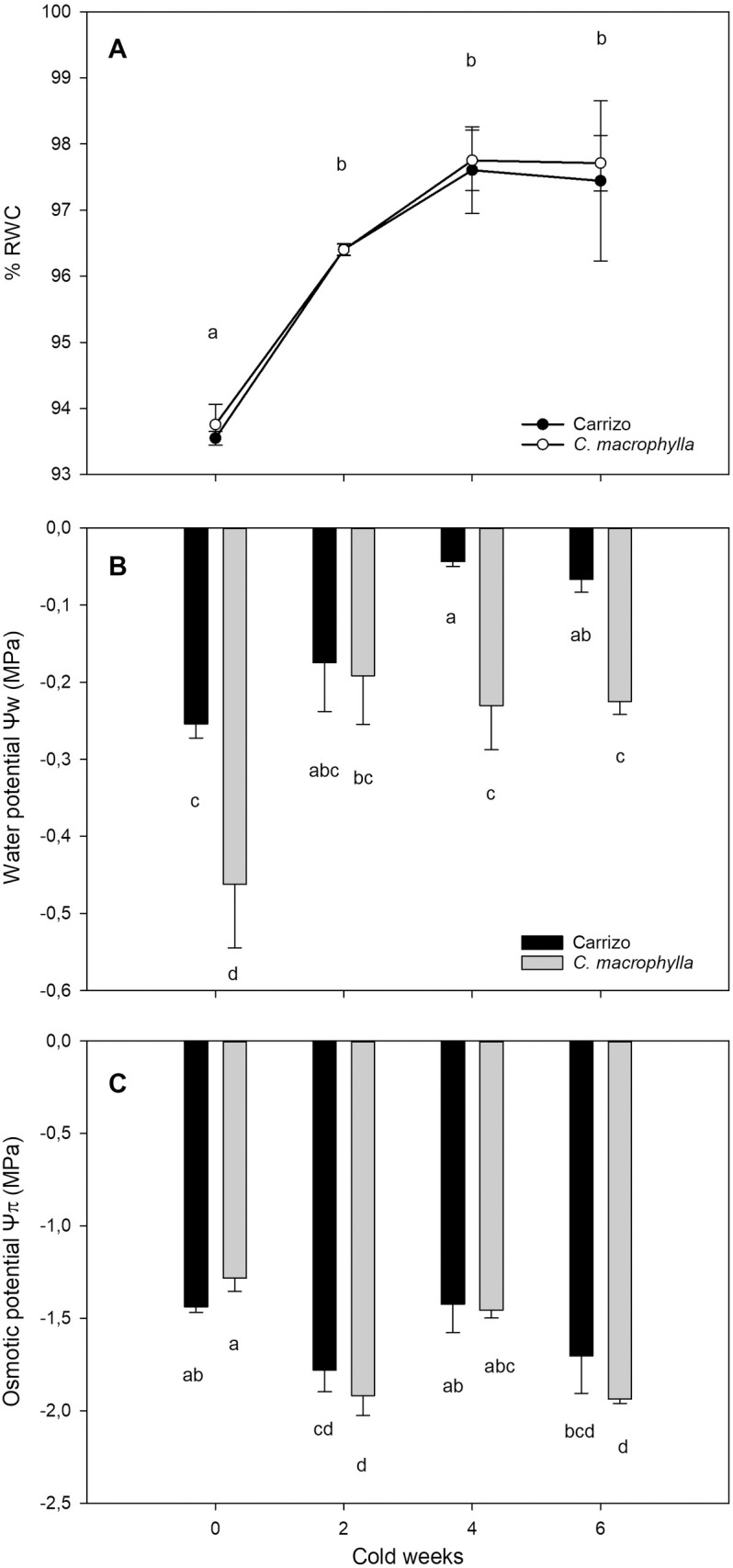
Measurement of water relations in the leaves. A) Relative water content, RWC% B) Water potential (ψW, MPa), C) Osmotic potential (ψπ, MPa). Measurements correspond to Carrizo citrange and *C. macrophylla* rootstocks grafted with Valencia delta seedless and grown at 1 °C for 0, 1, 2, and 6 weeks. The values shown represent the mean ± SD among six biological replicates (n = 6) and two technical replicates. The effect of the treatment over different times was tested using an LSD test from multiple ANOVA. The use of letters indicates significant differences, and the absence of a letter indicates ‘no significant difference’.

The relative water content in the leaf (RWC%) was quantified (Fig 2A). In both rootstocks, RWC% increased over time, becoming statistically significant after 6 weeks of low-temperature treatment with no differences between the rootstocks respect the initial/control week. This result was consistent with previous findings regarding physiology, both in photosynthesis and water potential, indicating that the plant is not translocating water over long distances from the soil to the leaves.

Water potential (ψw) measurements yielded unexpected results over the long term. Water potential (Fig 2B) increased with time, indicating that both genotypes halted apoplastic water transport and did not need to acquire water from the soil. This was consistent with previous results, where CO_2_ fixation, stomatal conductance, and evapotranspiration were fully repressed, significantly reducing the plant’s need for water uptake. An intrinsic difference was observed between the two genotypes, with each rootstock showing statistically different water potential values from the beginning of the treatment and maintaining these differences during cold treatment. Water potential increased over time to about half of its value in control plants, indicating no statistically significant differences between the rootstocks. ANOVA (S2 Table) showed a non‑significant genotype × time interaction (p = 0.2568), indicating that the cold‑induced increase in water potential was similar in magnitude between the two rootstocks.

Osmotic potential (ψπ) (Fig 2C) decreased over time in both rootstocks, from values of –1.4 MPa and –1.3 MPa in Carrizo citrange and *C. macrophylla*, respectively, in control plants, to –1.7 MPa and –1.8 MPa after 6 weeks of cold treatment. Osmolyte synthesis occurred in both rootstocks respect the initial/control week, although no significant differences between the rootstocks were detected during the cold stress.

### Determination of chlorophyll fluorescence during long-term cold stress

**Fig 3:**
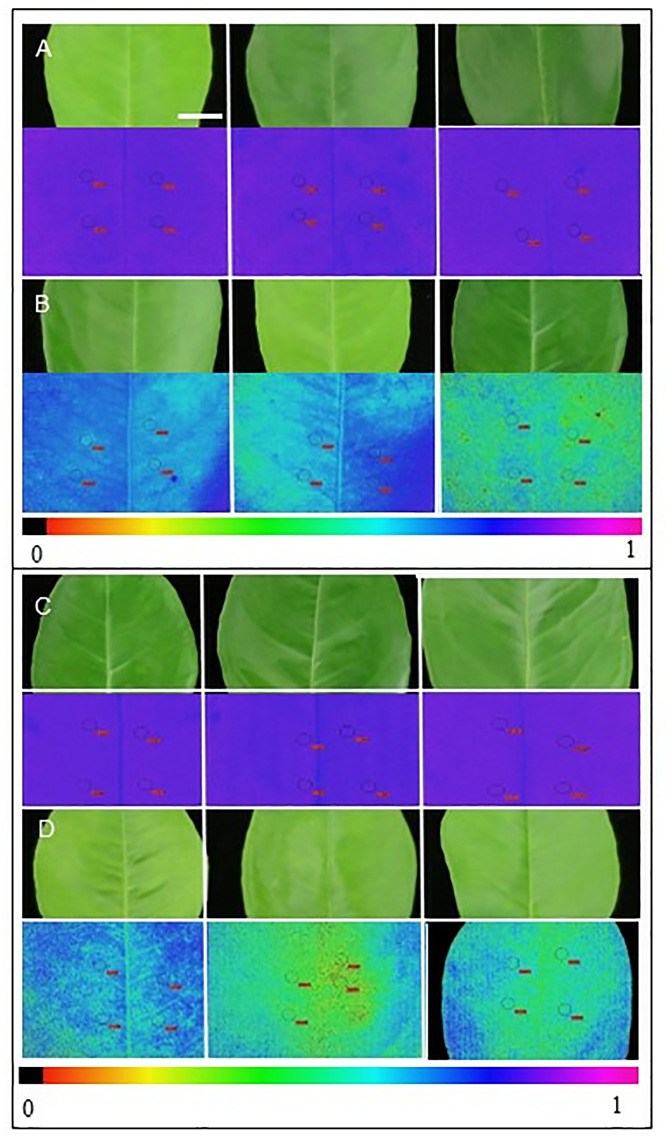
Maximum Quantum Yield of Photosystem II (Fv/Fm) and Leaf Images. A) Leaves of Carrizo citrange under control treatment. B) Leaves of Carrizo citrange rootstock after 6 weeks of treatment. C) Leaves of *C. macrophylla* under control treatment. D) Leaves of *C. macrophylla* plants after 6 weeks of treatment. The figure shows leaves of Carrizo citrange and *C. macrophylla* rootstocks grafted with Valencia delta seedless and grown at 1 °C for 0 and 6 weeks. The Fv/Fm values ranged from 0 (black color on the scale) to 1 (pink color). The white bar represents a 1 cm scale on the figure.

A study of chlorophyll fluorescence imaging (CFI) was conducted using a PAM-2100 Walz fluorimeter (Fig 3A-D). Fig 3 showed the representation of maximum quantum yield of photosystem II (Fv/Fm) values in colour and the corresponding leaf image. The photos reveal that the cold treatment did not cause visible damage to fully develop adult leaves (Fig 3A-D). However, the colour representation of Fv/Fm illustrated the damage caused by the temperature decrease over time. Table S1 presented the quantitative data at weeks 0, 2, 4, 6 of Fv/Fm and showed a decrease in the maximum efficiency of photosystem II. In this case, the Fv/Fm of the *C. macrophylla* rootstock was significantly lower than that of Carrizo citrange, 0.516 compared to 0.625 after 6 weeks of treatment, indicating that the PSII of *C. macrophylla* plants was more affected by the photoinhibition process than Carrizo plants.

### PIP aquaporins and water relations

**Fig 4:**
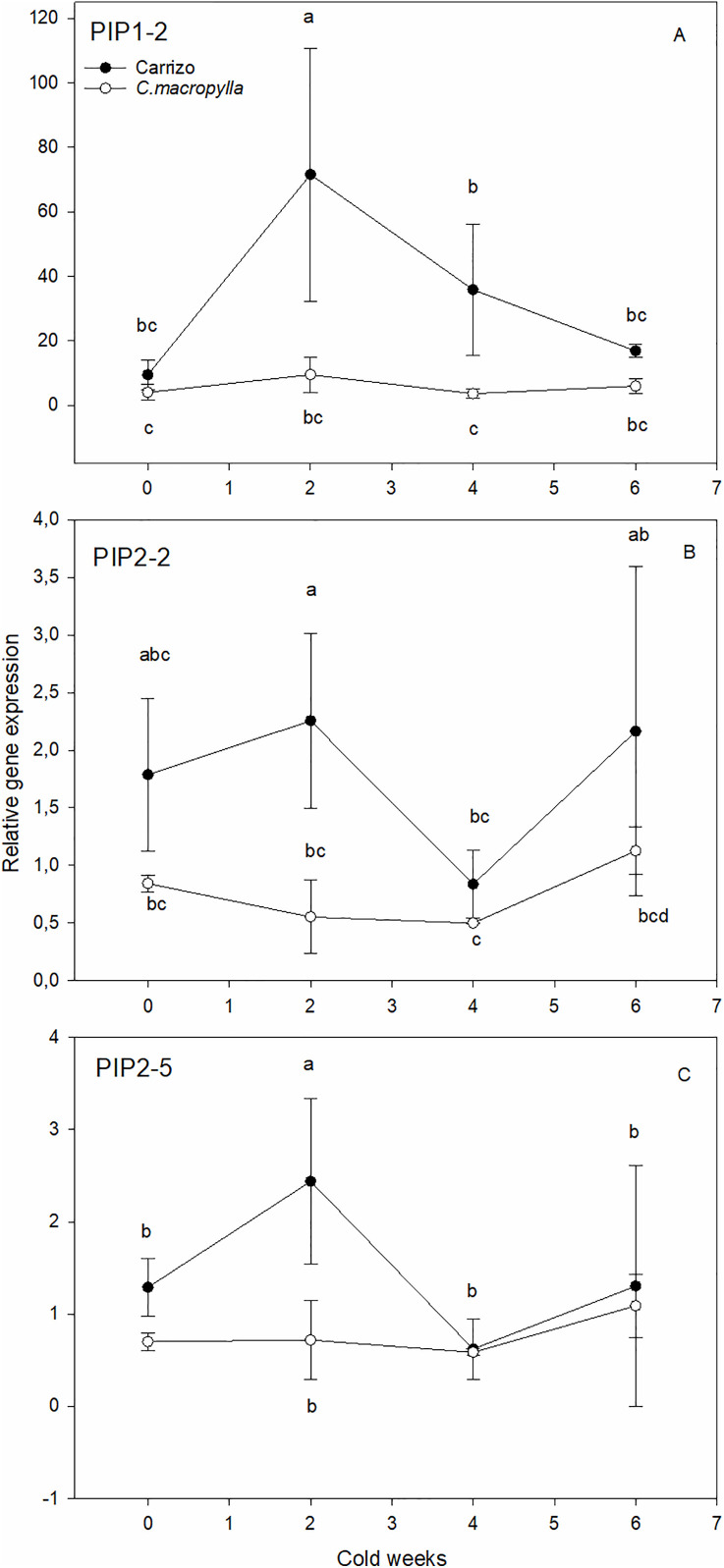
Relative gene expression of putative PIP genes selected in *C. macrophylla* and Carrizo citrange. Quantification of PIP aquaporins in the grafted rootstocks Carrizo citrange (CAR) and *C. macrophylla* (MAC) subjected to 1°C for 0, 2, 4 and 6 weeks. A) Putative *PIP1-2* gene (*LOC18044530*), B) putative *PIP2-2* gene (*LOC18039157*), and C) putative *PIP2-5* gene (*LOC18043954*). The values shown represent the mean ± SD among three biological replicates (n = 3) and three technical replicates. The effect of the treatment over different times was tested using an LSD test from multiple ANOVA.

As previously mentioned, in previous works [16], we realized a RNAseq assay with this samples from Carrizo citrange and *C. macrophylla* grafted into Valencia delta seedless variety under long-term cold temperatures. Based on this, we use three aquaporins potentially associated with long-term cold tolerance. Consequently, Fig 4 showed the gene expression of the following aquaporins: PIP1–2 (LOC18048648), PIP2–1 (LOC18035633), and PIP2–5 (LOC18034159). The gene encoding the putative aquaporin PIP1–2 exhibited a significant 8‑fold increase in expression at 2 weeks relative to its respective control, and a 31‑fold increase compared to plants grafted onto *C. macrophylla* (Fig 4A). Following 4 and 6 weeks of cold treatment, expression levels in plants grafted onto Carrizo citrange rootstock declined, remaining 40‑fold and 20‑fold higher than in *C. macrophylla*, respectively. In *C. macrophylla* plants, *PIP1‑2* expression did not change or was not induced at any of the sampling time points. Expression of the putative *PIP2‑2* gene increased in Carrizo citrange plants at 2 weeks of treatment relative to *C. macrophylla*; however, by 4 weeks it had decreased compared to its own control (Fig 4B). After 6 weeks, expression levels in both rootstock combinations did not differ significantly from their respective controls. In Carrizo citrange grafted plants, *PIP2‑5* expression was upregulated more than threefold at 2 weeks (Fig 4C), whereas in *C. macrophylla* plants no significant differences were observed among the time points.

Thus, we found that the three aquaporins (Fig 4A-C) were more highly expressed at 2 weeks of treatment in Carrizo citrange plants compared to *C. macrophylla* plants, highlighting the expression of the aquaporin PIP1–2 after two weeks of cold.

## Discussion

### Photosynthesis and water relations under long-term cold stress conditions

The water balance initially appeared somewhat contradictory, as a decrease in water potential, and consequently in RWC% and stomatal closure, was expected, like what occurs in other crops. This would indicate a combined physiological response to drought and cold [30,31]. However, according to the results, both Carrizo citrange and *C. macrophylla* plants showed an increase in water potential, which was more negative in *C. macrophylla*. Osmotic potential slightly decreased, becoming more negative after 4 weeks of cold exposure in both genotypes, while RWC% remained like the control plants. Consequently, photosynthesis ceased. According to our results and as observed in other crops, some parameters can be interpreted as if the plant were experiencing severe drought, such as photosynthesis, stomatal conductance, transpiration, and internal CO_2_ [32,33]. Nevertheless, other parameters, such as water potential, RWC%, and even osmotic potential, remained at values indicative of good irrigation and active plant growth. In citrus, several studies have reported similar findings where water potential and RWC% at cold stress did not change in *C. sinensis* plants grafted onto Carrizo citrange rootstock [34,35]. Similarly, in *Vitis vinifera*, treatment at temperatures of 4, 0, and −4 °C had little effect on RWC% [36].

Regarding water relations, symplastic, apoplastic, and transcellular water transport are crucial for water balance in plants. Plants are an open system where water is incorporated through the roots, transported to different organs and tissues, and used for physiological functions through the three pathways. In this case, the cessation of photosynthesis and stomatal closure may result in the plant completely halting water consumption, thus not requiring water uptake from the soil. Under low-temperature conditions, plant metabolism, vegetative growth, and all cellular reactions are significantly reduced, thereby greatly diminishing water consumption. Moreover, it is known that 99.5% of the water within the plant is lost during transpiration, so if this process is inhibited along with photosynthesis, water loss is minimal. In fact, to fix 1 kg of carbon during photosynthesis, plants transport several hundred kilograms of water [37].

### The role of PIP aquaporins in long-term cold stress in citrus

Carrizo citrange maintain significantly less negative water potential values than *C. macrophylla* throughout the experiment (main effect of genotype, p-value = 0.0007). These results suggest that the greater cold tolerance of Carrizo is not due to a differential physiological response to stress, but rather to a more favourable basal water status, possibly associated with higher root hydraulic conductance and lower water loss, or a root architecture that facilitates water uptake. In contrast, *C. macrophylla*, consistently exhibiting more negative water potential values, is placed in a situation of greater hydraulic vulnerability during low-temperature stress, which would account for its sensitive character [17,38,39]. As mentioned, apoplastic water transport is crucial for maintaining cellular turgor. This transport primarily occurs thanks to MIPs, specifically PIPs. Three aquaporins (*PIP1–2*, *PIP2–2*, and *PIP2–5*) were overexpressed in Carrizo citrange (Fig 4). PIPs have been associated with tolerance to abiotic stresses such as salinity, drought, and low temperatures [40–42]. Although their molecular mechanism is unknown, aquaporins are known to play a significant role in cellular water transport.

As previously mentioned, the cellular membrane system is the first organelle affected by decreasing temperatures in plants [43]. It is well established that tissue freezing causes membrane damage, leading to dehydration [44,45]. When temperatures drop below 0 °C, ice formation begins in the intercellular spaces partly because the extracellular space has a higher freezing point due to a lower solute concentration than the intracellular fluid [46]. As the chemical potential of ice is lower than that of liquid water at a given temperature, extracellular ice formation results in a decrease in water potential outside the cell, causing unfrozen intracellular water to move to the intercellular space. At −10 °C, more than 90% of osmotically active water moves out of the cells, and the osmotic potential of the remaining unfrozen fluid usually reaches 5 osmolar [46]. Thus, in general terms, plants experiencing cold stress also undergo cellular dehydration stress despite water availability in the soil. It is thought that certain PIPs that are overexpressed at low temperatures may function at the cellular level by increasing membrane permeability to reduce intracellular dehydration and mechanical stress [47,48].

As we have observed in the present work in citrus, it also occurs in other species where the repression or overexpression of PIPs led to increased tolerance to cold temperatures. In banana plants overexpressing *MusaPIP1–2* and *MusaPIP2–1*, increased drought and cold tolerance were observed [49]. Tobacco plants overexpressing *TaAQP7* (*PIP2*) also showed increased tolerance to cold and drought [50]. In *A. thaliana*, *AtPIP1–4* and *AtPIP2–5* work together to regulate cold response and acclimation [42].

Additionally, in *C. macrophylla* plants, where the DEGs at 2 weeks compared to Carrizo citrange plants are shown, a specific decrease in the genes encoding *PIP2*s was observed. *PIP1*s and *PIP2*s have certain functional differences. Besides the diacidic motif in the N-terminal position of PIP2s, *PIP1* aquaporins must heteromerize with *PIP2* aquaporins to be fully functional. This is demonstrated by FRET interaction analysis studies [51] with oocytes where maize proteins *ZmPIP1–2* and *ZmPIP2–1* co-purify [52] and studies in *Beta vulgaris* showing that *PIP1* and *PIP2* form heterotetramers [53]. Mathematical models based on stoichiometric relationships also correlate RNA ratios in strawberry for *FaPIP1–1* and *FaPIP2–1* with a *FaPIP2–1* mutant [54]. Thus, this co-expression suggests that in Carrizo citrange plants, some combinations of *PIP1* and *PIP2* (*PIP1–2*, *PIP2–1*, and *PIP2–5*) might be expressed. Moreover, in *C. macrophylla* plants, the exclusive overexpression of *PIP1–2* suggests a minimal effect since *PIP1* alone would have very low activity in cold stress tolerance, thereby reducing apoplastic water transport and cause dehydration at the cellular level. Additionally, in *C. macrophylla* plants, the significant decrease in PIPs might be causing a slight decrease in water potential compared to Carrizo citrange.

Overall, aquaporins are considered to play an important role in acclimation and tolerance to cold stress, which are two independent phenomena. Cold stress tolerance involves preventing freezing damage to the plasma membrane, while the acclimation process, which increases cold tolerance, minimizes plasma membrane injury. Therefore, it is suggested that the *C. macrophylla* genotype takes longer to reach the acclimation process, making it more vulnerable to low-temperature stress [16]. Nonetheless, further experiments related to PIPs should be conducted to conclude these results.

## Conclusions

Long-term cold stress in the Valencia deltaseedless variety grafted onto Carrizo citrange and *C. macrophylla* rootstocks resulted in a halt in photosynthesis, stomatal closure, reduced transpiration, and an accumulation of internal CO_2_, indicating that both genotypes are experiencing significant stress and are attempting to survive the temperature drop. The water balance of citrus plants in response to cold temperatures often behaves in a way that may seem contradictory. Cold temperatures lead to stomatal closure, reduced transpiration, and a significantly decreased osmotic potential (Ψπ), like what occurs under conditions of extreme drought, as the plant seeks to prevent water loss through transpiration. However, the stem water potential (Ψw) and relative water content (RWC%) often remain at levels typical of actively growing plants under adequate irrigation conditions, similar to what is observed during warm, sunny days. In this context, it is important to consider the transcellular water movement that occurs through aquaporins. However, gene expression data suggest that plants grafted onto Carrizo citrange rootstock, may contribute to the better acclimation and tolerance under cold stress leading to the overexpression of aquaporins *PIP1–2*, *PIP2–2*, and *PIP2–5.*

## Supporting information

S1 Table Maximum Quantum Yield of Photosystem II, Fv/Fm values in arbitrary units. Measurements correspond to Carrizo citrange and *C. macrophylla* rootstocks grafted with Valencia delta seedless and grown at 1 °C. Fv/Fm measurements were taken at 0, 2, 4, and 6 weeks. The values shown represent the mean ± SD among three biological replicates (n = 3) and two technical replicates. The effect of the treatment over different times was tested using an LSD test from multiple ANOVA. The use of letters indicates significant differences, and the absence of a letter indicates ‘no significant difference’.
(DOCX)

S2 Table Results of the multifactorial ANOVA: interactions of leaf water potential (Ψw, MPa) for *Citrus macrophylla* and Carrizo citrange rootstocks grafted with Valencia delta seedless and grown at 1 ºC over time, 0, 2, 4, and 6 weeks. F-ratios are based on the mean square of the residual error.
(DOCX)

S1 Data Raw Data. (XLSX)
