# Supplementary material for: Response of PIP aquaporins to long-term cold stress in two citrus rootstocks
Source: PLoS One. 2026 Jul 31;21(7):e0355050. doi: 10.1371/journal.pone.0355050 (PMC13426935; doi:10.1371/journal.pone.0355050)
Supplement: S1 Table — Measurements correspond to Carrizo citrange and C. macrophylla rootstocks grafted with Valencia delta seedless and grown at 1 °C. Fv/Fm measurements were taken at 0, 2, 4, and 6 weeks. The values shown represent the mean ± SD among three biological replicates (n = 3) and two technical replicates. The effect of the treatment over different times was tested using an LSD test from multiple ANOVA. The use of letters indicates significant differences, and the absence of a letter indicates ‘no significant difference’. (DOCX) [file pone.0355050.s001.docx]

**S1 Table. Maximum Quantum Yield of Photosystem II, Fv/Fm values in arbitrary units**. Measurements correspond to Carrizo citrange and *C. macrophylla* rootstocks grafted with Valencia delta seedless and grown at 1 ºC. Fv/Fm measurements were taken at 0, 2, 4, and 6 weeks. The values shown represent the mean ± SD among three biological replicates (n=3) and two technical replicates. The effect of the treatment over different times was tested using an LSD test from multiple ANOVA. The use of letters indicates significant differences, and the absence of a letter indicates ‘no significant difference’.

| Cold weeks | Carrizo | SD | *C. macrophylla* | SD |
| --- | --- | --- | --- | --- |
| 0 | 0.810 | 0.00 ab | 0.814 | 0.00 a |
| 2 | 0.701 | 0.03 bc | 0.668 | 0.01 c |
| 4 | 0.592 | 0.08 cd | 0.594 | 0.02 cd |
| 6 | 0.625 | 0.02 c | 0.516 | 0.05 d |
