# Supplementary material for: Response of PIP aquaporins to long-term cold stress in two citrus rootstocks
Source: PLoS One. 2026 Jul 31;21(7):e0355050. doi: 10.1371/journal.pone.0355050 (PMC13426935; doi:10.1371/journal.pone.0355050)
Supplement: S2 Table — F-ratios are based on the mean square of the residual error. (DOCX) [file pone.0355050.s002.docx]

**S2 Table**. **Results of the multifactorial ANOVA**: interactions of leaf water potential (Ψw, MPa) for *Citrus macrophylla* and Carrizo citrange rootstocks grafted with Valencia delta seedless and grown at 1 ºC over time, 0, 2, 4, and 6 weeks. F-ratios are based on the mean square of the residual error.

| *Source* | *Sum of Squares* | *Main Square* | *F-ratio* | *P-value* |
| --- | --- | --- | --- | --- |
| Main effects |  |  |  |  |
| A: genotype | 0.129957 | 0.129957 | 16.44 | **0.0007** |
| B: time | 0.216278 | 0.0720927 | 9.12 | **0.0007** |
| INTERACTIONS |  |  |  |  |
| AB | 0.0348137 | 0.0116046 | 1.47 | **0.2568** |
| RESIDUALS | 0.142329 | 0.00790714 |  |  |
| TOTAL (corrected) | 0.582002 |  |  |  |
